# Laser-Induced Graphene Microsupercapacitors: Structure, Quality, and Performance

**DOI:** 10.3390/nano13050788

**Published:** 2023-02-21

**Authors:** Andres Velasco, Yu Kyoung Ryu, Assia Hamada, Alicia de Andrés, Fernando Calle, Javier Martinez

**Affiliations:** 1 Instituto de Sistemas Optoelectrónicos y Microtecnología, Universidad Politécnica de Madrid, Av. Complutense 30, 28040 Madrid, Spain; 2 Departamento de Ingeniería Electrónica, Escuela Técnica Superior de Ingenieros de Telecomunicación, Universidad Politécnica de Madrid, Av. Complutense 30, 28040 Madrid, Spain; 3 Instituto de Ciencia de Materiales de Madrid, Consejo Superior de Investigaciones Científicas, C/Sor Juana Inés de la Cruz 3, Cantoblanco, 28049 Madrid, Spain; 4 Departamento de Ciencia de Materiales, Escuela Técnica Superior de Ingenieros de Caminos, Canales y Puertos, Universidad Politécnica de Madrid, C/Profesor Aranguren s/n, 28040 Madrid, Spain

**Keywords:** graphene, supercapacitor, laser-induced graphene, energy storage, specific capacitance

## Abstract

Laser-induced graphene (LIG) is a graphenic material synthesized from a polymeric substrate through point-by-point laser pyrolysis. It is a fast and cost-effective technique, and it is ideal for flexible electronics and energy storage devices, such as supercapacitors. However, the miniaturization of the thicknesses of the devices, which is important for these applications, has still not been fully explored. Therefore, this work presents an optimized set of laser conditions to fabricate high-quality LIG microsupercapacitors (MSC) from 60 µm thick polyimide substrates. This is achieved by correlating their structural morphology, material quality, and electrochemical performance. The fabricated devices show a high capacitance of 22.2 mF/cm^2^ at 0.05 mA/cm^2^, as well as energy and power densities comparable to those of similar devices that are hybridized with pseudocapacitive elements. The performed structural characterization confirms that the LIG material is composed of high-quality multilayer graphene nanoflakes with good structural continuity and an optimal porosity.

## 1. Introduction

Supercapacitors are energy storage devices that are classified into three main groups: electrochemical double-layer capacitors (EDLCs), pseudocapacitors, and hybrid capacitors [1,2]. In the case of EDLCs, the ionic charges of the electrolyte are arranged as a double layer (as modeled by Gouy, Chapman, and Stern), contributing to the charge storage of the device. This simple principle—which does not involve chemical reactions, only non-faradaic charge processes—allows for fast kinetics, greater cyclability, and a typically higher power performance than batteries [3]. EDLC electrodes must be highly conductive and have a high specific surface area through a micro- and/or nano-porous network, which is accessible to the electrolyte. Graphene, carbon nanotubes (CNTs), and carbon nanofibers are some of the most promising materials to use for supercapacitors’ electrodes due to their high electrical conductivity and excellent mechanical flexibility [4,5,6]. In addition to these properties, graphene presents the largest specific surface area and, consequently, a great deal of research has been conducted in trying to optimize its properties and microstructure for this application [7,8,9].

Among the several available fabrication methods [10], laser-induced graphene (LIG) by direct laser writing has attracted a lot of attention since its proposal in 2014 [11,12]. This is because it presents several attractive features, such as: it is a fast process, which simultaneously enables material transformation and device patterning in a single step, and it is low cost and does not require cleanroom conditions. In this process, a laser beam is moved across the surface of a solid precursor to transform the material properties point by point, producing carbonaceous, three-dimensional, and porous graphene-like material [13,14,15]. Another strength of the technique is its high tunability: The laser wavelength, the scribing conditions, and the precursor material [16,17,18,19] influence the morphology [20,21,22] and properties of the produced LIG material [23]. Therefore, by selecting different combinations of those parameters [24], different LIG materials can be produced for a variety of applications ranging from sensors to heating devices [25,26,27,28,29,30,31,32], including the main focus of this paper—supercapacitors [33]. In Table S1 (Supplementary Materials), a summary of the common and specific traits, advantages, and disadvantages of the available laser methods are displayed.

The most used precursor to fabricate LIG, and the focus of this work, is polyimide, which is also referred to by its commercial name, Kapton. This polymer generates high-quality porous carbons upon laser exposure, mostly due to its highly aromatic nature [11,34,35]. Furthermore, it has a low thermal expansion, allowing it to generate micrometer-sized pyrolized patterns on it without excessive geometrical deformation. The vast majority of research groups use commercial Kapton with a thickness of 125 µm. We studied the properties of the LIG while it was fabricated in a 60-micron-thick Kapton, since thinner films are better for flexible applications—as several recent works have pointed out [13,21,36,37,38,39,40]. Our study provides an understanding on the correlation between morphology, material quality, and electrochemical performance in order to find the optimal laser parameters for the fabrication of LIG microsupercapacitors on thin Kapton substrates. The electrochemical characterization revealed a high capacitance of 22.2 mF/cm^2^ at 0.05 mA/cm^2^, as well as energy and power densities that are better than those of similar devices, which are fabricated on 125 μm thick Kapton.

## 2. Materials and Methods

LIG MSC samples have been fabricated by applying a high-power CO_2_ laser beam to the Kapton substrate. The samples will be named using the preparation parameters “S-laser power (W)—scan speed (mm/s)”. Both the power of the laser and its scan speed were varied in a narrow laser fluence range that kept the structural stability of the transformed LIG material, thus meaning a continuous surface and without gaps, cracks, or other structural deficiencies. This balance is difficult to achieve, but is key to transform a very thin solid polymer layer into a porous carbon network that is structurally stable, electrically conductive, and with good graphenic quality, as well as adequate porosity and morphology. To evaluate the correlation between the structure, quality, and performance, scanning electron microscopy (SEM), Raman spectroscopy mapping, and electrochemical characterization, in relation to supercapacitors, were carried out. Regarding the electrochemical characterization, the key metrics of supercapacitors are its capacitance, energy density, and power density [41,42], which may be normalized by the active weight, volume, and/or area of the electrodes. In the case of planar interdigitated microsupercapacitors (MSC), the area is the normalizing factor of choice and the one used here.

### 2.1. Laser Pyrolysis

A commercial, low-cost, hobby-grade laser cutter, equipped with a 40 W continuous wave infrared CO_2_ laser of 10.6 µm wavelength, and an X-Y computer control system was used in this paper to transform a 60 µm thick polyimide film (Tesa^®^, Norderstedt, Germany), which was coated with silicone adhesive, into the LIG material. The designs were created with a vector image editing software (Inkscape), sent to the laser through a software, and engraved by moving the laser continuously in the x direction; this was conducted with a y-line spacing ($d_{y}$) of 75 µm. The focused beam size (s) was measured to be around 100 µm, which is in agreement with the literature [11]. This ensures full coverage while in raster mode, engraving the designs bottom-up in order to avoid dust buildup. The minimum y-distance separation, or resolution that the machine can achieve is 25 µm. Laser power can range from 1.8 W up to the maximum 40 W, and the scan speed can reach up to 600 mm/s.

A parameter that is often referred to in the literature is the laser fluence, which is a measure of the areal energy that is irradiated to a surface (in J/cm^2^). This is given by the formula [13]:

$$Fluence=\frac{P}{u_{x}\cdot s}\times \frac{s}{d_{y}}$$

where P is the laser power in watts, $u_{x}$ is the lateral scan speed of the laser, s is the laser pitch or diameter, and $d_{y}\;$the step interval between lines. The product $u_{x}\cdot s$ can be understood as a focal spot moving along the *x*-axis: the faster it moves, the lower the fluence. The multiplier $\frac{s}{d_{y}}$ is the overlapping factor of the energy that is irradiated to a laser line as $\;s>d_{y}$.

### 2.2. MSC Fabrication

**Figure 1:**
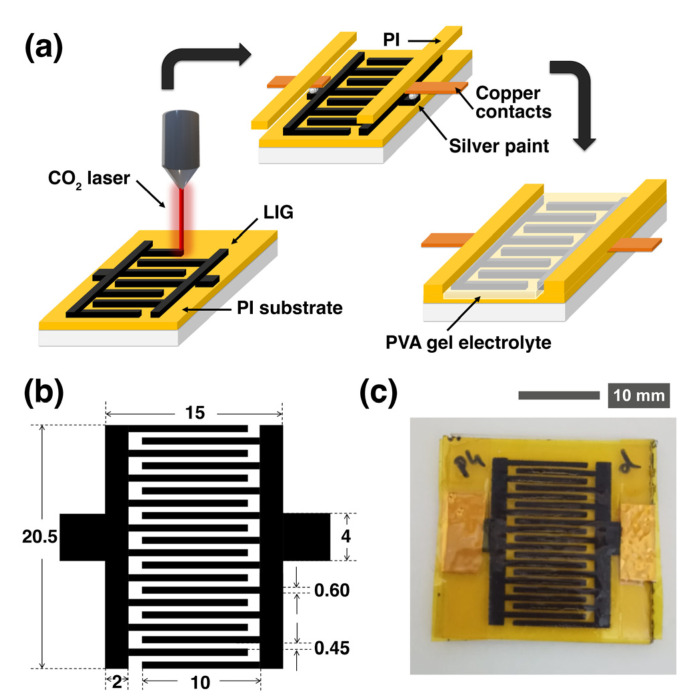
(**a**) Schematic drawing of the laser setup that was constructed to irradiate the polyimide. The LIG electrodes were electrically contacted with silver paint and copper, as well as, finally, the PVA gel electrolyte. (**b**) Top view of the computer design used to fabricate the devices (dimensions in mm). (**c**) Photograph of the final fabricated device after all the processing steps.

The process is described graphically in Figure 1a. A 60 µm thick polyimide (PI) film is fixed onto a clean microscope glass slide to avoid trapping air pockets below. Then, an interdigitated MSC design, such as the one shown in Figure 1b, is lasered onto the PI film—working as both the active electrode and current collector. Each fabricated electrode is contacted with an adhesive copper tape, reinforcing the contact through a drop of silver paint (the tape and paint are from RS PRO). In the following step, the metallic contacts are covered using an adhesive PI film to protect them from the acid gel electrolyte. A droplet of it is applied and then spread onto the active area of the device. In the next step, the sample is placed under an active vacuum for 2 h; this is performed to maximize the removal of air from the internal pores, thus replacing it with electrolyte. Finally, the device is left in a static vacuum overnight to ensure a good infiltration of the electrolyte into the smallest pores.

The poly (vinyl alcohol) (PVA):H_2_SO_4_ 1M gel electrolyte was fabricated by using a standard procedure [43], which involved heating a 1M acid solution at 80 °C and adding 1 g of 86–88% hydrolyzed, medium molecular weight PVA beads that were previously crushed manually in a mortar, per 10 mL of acid solution, while mechanically stirring via a magnetic bead in order to avoid agglomeration. This was performed without introducing air into the gel. After 2 h of heating and stirring, when the solution becomes clear and viscous, it was left to cool down at room temperature to allow the bubbles to rise.

### 2.3. Material Characterization

The electrical resistance of the samples was tested by measuring via the two probes method. This was performed by using a probe station (Karl Suss PSM 6) and a parameter analyzer (4145B, Hewlett-Packard). For each of the laser conditions used in this work, three 4 mm wide and 20 mm long LIG strips were measured in order to provide an average value with the corresponding standard deviations.

A Scanning Electron Microscope (SEM) (FEI inspect F50), while using an accelerating voltage of 5 kV, was used to study the morphology of the produced LIG electrode materials. Eight 4 × 4 mm^2^ LIG square areas were fabricated onto the 60 µm thick Kapton. In this work, this was conducted once for each pair of laser conditions (power and scan speed). An electrical path was added to avoid charging effects by using conductive adhesive copper tape and silver paint, as explained above. Images at four different magnifications were taken for each sample to study the homogeneity and the porous morphology, as well as to estimate the average pore size. For the electrochemical study that was used in this work, between three and five samples of planar interdigitated LIG microsupercapacitors were used and manufactured for each condition in order to achieve better statistics and to minimize the dispersion of the results.

Micro-Raman experiments were performed using the 488 nm line of an Ar^+^ laser with an incident power of 7 mW. An Olympus microscope with a ×20 objective and with a high optical aperture (N.A. = 0.95) allowed for a <0.8 μm lateral resolution. The scattered light was filtered with a notch filter (Kaiser) and analyzed with a Horiba (iHR-320) monochromator (1200 L/mm grating), which was coupled to a Peltier cooled Synapse CCD. Raman spectra were obtained along two lines of 150 µm long with 5 µm steps and 20 µm spacing.

Pristine graphene presents two main peaks, G and 2D, at around 1580 and 2700 cm^−1^, respectively. This was conducted with an I_2D_/I_G_ ratio, typically in a range from 2 to 6, which was reduced to around 0.5 for the multilayer graphene. The presence of point defects or edges promotes the defect D peak at around 1340 cm^−1^. To obtain the I_D_/I_G_ and I_2D_/I_G_ ratios, as well as the full widths at the half maximum (FWHM) of the peaks, the G peak was fitted with an asymmetric Breit–Wigner–Fano (BWF) function, while for the D and 2D peaks, Lorentz functions were used.

Regarding the electrochemical measurements, a potentiostat/galvanostat system (Autolab PGSTAT204) was used to perform the cyclic voltammetry (CV), galvanostatic charge–discharge (GCD), and electrochemical impedance spectroscopy (EIS) measurements on the finished devices; these measurements were conducted by using the software “Nova”. The device areal capacitance (*C_area_*, in mF/cm^2^) was extracted from the GCD measurements though the formula [44]:

Carea=IdischargeS×dVdt

where *I_discharge_* is the constant discharge current, *S* is the total area of both positive and negative electrodes, and *dV/dt* is the slope of the discharge curves.

The areal energy density (in µWh/cm^2^) is calculated through the formula:

$$E_{area}=\frac{1}{2}\times C_{area}\times \frac{{\left(\Delta V\right)}^{2}}{3600}$$

where $\Delta V=V_{max}-V_{drop}$ is the discharge voltage, being *V_max_* = 1 V for aqueous electrolytes, and *V_drop_* is the voltage difference between the *V_max_* and the next point in the discharge curve.

Finally, the areal power density (in mW/cm^2^) is given by:

$$P_{area}=\frac{E_{area}}{\Delta t}\times 3600$$

where Δt is the discharge time (in seconds).

## 3. Results

**Figure 2:**
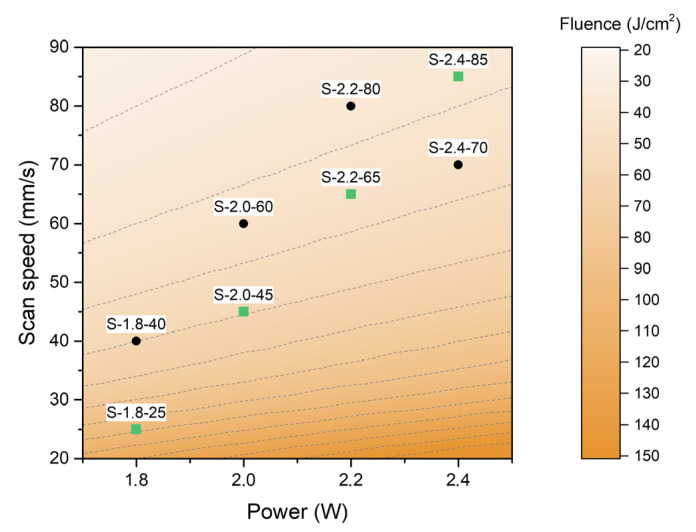
Laser power (W) versus scan speed (mm/s) with the eight chosen laser conditions plotted on top. In the background, a color map of the laser fluence is calculated with a scan line width of 75 um.

Figure 2 shows the eight chosen laser conditions that were used to irradiate the PI substrates. Both power and scan speed must be carefully chosen in order to maintain the structural integrity of the material, but must also be enough to achieve sufficient pyrolysis to transform the Kapton film substrate into a conductive 3D carbon network. Due to this balance, the samples have been fabricated in a concrete “fluence region”, as can be appreciated in the figure, by using the power and speed values that are the most adequate according to the observed results. For the laser conditions that were toward the top left corner, the laser power was found to be too weak and the scan speed was too fast to produce any noticeable change in the substrate or in regard to leaving large gaps that would break the continuity of the material. Moving toward the bottom right corner, the laser power becomes too strong and the scan speed too slow to keep the integrity of the pyrolyzed polymer, thus resulting in internal cracks and poor material quality. To help the readers, the values of power, scan speed, and fluence of the 8 samples of study are summarized in Table 1.

Apart from laser power and scan speed, the laser fluence calculation shown in the background of Figure 1 corresponds to a scan line width of 75 µm, which has been kept constant through all the experiments. This distance is smaller than the laser beam diameter, thus assuring the homogeneous irradiation of the whole material in order to pyrolyze the Kapton film and to form a continuous graphene electrode, which occurs with a slight overlap between the laser lines. In this work, the focal length used in these experiments was 8 mm and was kept constant.

In Figure 2 and Table 1, the eight different conditions studied in the present work are shown. For the presentation of the morphology, the quality of graphenic material, and the electrochemical characterization results, we have focused on the four conditions that are marked in green in Figure 2 and are marked with (*) in Table 1 in order to ease the visualization and comparison of the obtained materials to the readers. These conditions fall in a line where both the power and scan speed are spaced equally (0.2 W and 20 mm/s between samples).

As a first step, we checked the electrical resistance of the eight LIG samples that are considered in this work, as described in Table 1. Each sample was fabricated as a 4 mm wide and 20 mm long strip. We measured the resistance of three strips for each condition in order to take into account the sample-to-sample variation. Therefore, we provide the mean value of the three strips with the corresponding standard deviations, which are represented as error bars in the *y*-axis in Figure S1 (see Supplementary Materials (SM)). The set of the S-1.8-40 samples was not represented in the graph because they were not conductive, thus showing a very high resistance in the order of GΩ. We associate this result with the incomplete graphitization of the polyimide under 1.8 W and 40 mm/s, which leaves non-modified areas that are insulating, thus affecting the connectivity of the LIG. The set of the S-1.8-25 samples presented a resistance of 351 Ω and a high standard deviation. Under these conditions, the graphitization level was enough to ensure the connectivity of the material, but still not enough to achieve the graphene with the best quality. The set of the S-2.0-45 samples presented the lowest resistance, around 116 Ω, and with the smallest standard deviation. The sets of the samples made under the rest of the conditions presented resistances in the range of 133–156 Ω, with higher standard deviations. On the basis of these results, we conclude that the conditions that produce the most conductive and reproducible LIG are a power of 2 W and a scan speed of 45 mm/s. Macroscopically, we correlate this highest conductivity with a better connectivity of the pores in the LIG network and the optimal level of graphitization [13,21], which will be corroborated later with the other characterization techniques.

**Figure 3:**
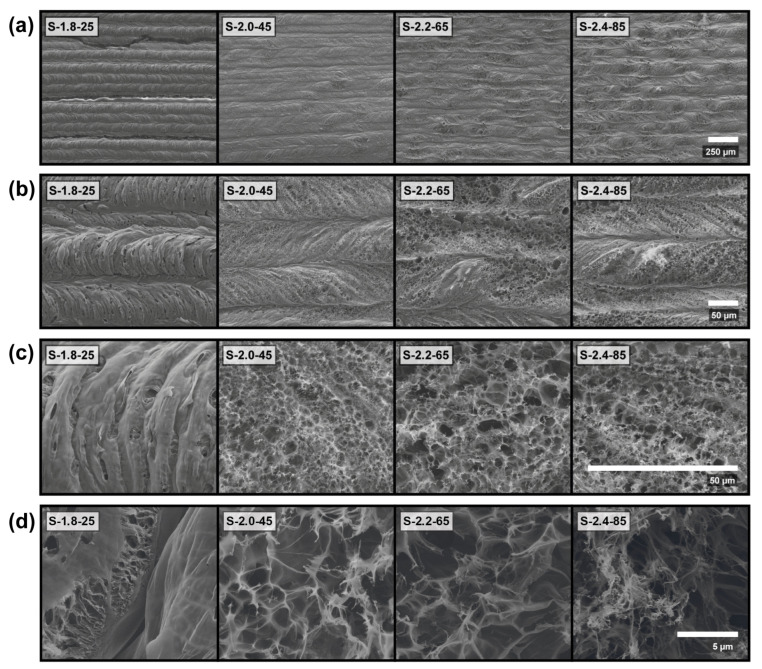
SEM images of the four LIG samples, at four different magnifications. Magnifications of 200× (**a**), 1000× (**b**), 5000× (**c**) and 20,000× (**d**). The scale is the same for all images in the same row and is given at its bottom right corner.

Figure 3 shows SEM images of the four LIG samples of interest, at four different magnifications. The sample S-1.8-25, produced at the lowest laser power, was graphitized with lower degassing, thereby producing a continuous material that showed very few open pores (Figure 3d). In Figure 3b,c, the laser movement direction can be appreciated as the polymer substrate expands and bends when the laser passes, creating characteristic C-shaped lines when the laser is moving left to right, and the reverse for the opposite direction. The same can be seen with the S-shaped diagonal ripples for higher power samples. For laser powers of 2 W and higher, the porosity was greatly increased. In sample S-2.0-45, its porosity was the most uniform and with the smallest pore size of around 2.5 µm, as can be seen in Figure 3c,d. This sample was also the densest, achieving a good compromise between the generated porosity and a, non-excessive, material loss. Samples made with laser powers higher than 2 W, as Figure 3b shows, present bigger pores and gaps. This is because the removal of the material during the laser induction process was much more pronounced. At the highest magnification (Figure 3d), the third sample, S-2.2-65, fabricated through a higher laser power, shows a more heterogeneous porous network with bigger pores in the range of 4 µm. Sample S-2.4-85 shows a very similar structure, but at the lowest magnification (Figure 3a) the heterogeneity of the sample is even higher, as the degradation induced by the laser collapsed the pores, thus transitioning its morphology into strings and reducing its effective surface area (Figure 3d).

For each region, we obtained Raman spectra along two lines of 150 µm long (30 spectra in each), which were separated by 20 µm instead of by single point measurements because the LIG formation process has an inhomogeneous nature due to the intensity dispersion of the impinging laser beam; this phenomenon is typically described as a Gaussian function through its radius [21]. Raman mapping allows us to observe the level of transformation and its variation as a function of the position, thereby gathering information about this inhomogeneity that, we believe, is key to understand the macroscopic properties of this material. This mapping reflects a collection of high quality parallel LIG lines, which are connected via lesser quality regions in between them.

**Figure 4:**
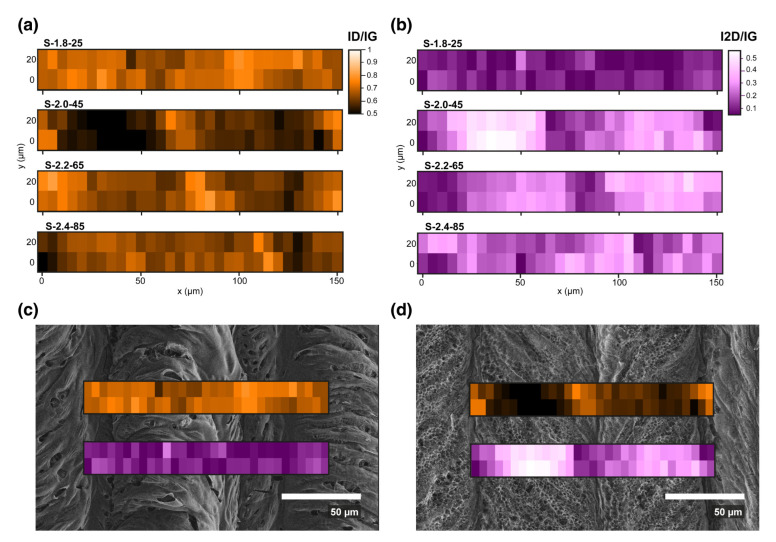
Raman spectroscopy mapping of the regions of each LIG sample (20 by 150 µm), covering several laser lines (samples have been rotated 90° to fit the equipment). (**a**) Raman peak intensity ratio I_D_/I_G_ and (**b**) Raman peak intensity ratio I_2D_/I_G_. (**c**,**d**) present the I_D_/I_G_ and I_2D_/I_G_ maps that were overlapped to the corresponding SEM image of samples S-1.8-25 and S-2.0-45, respectively. The overlapping shows a good spatial correspondence between the stripped and periodic nature of the writing process (SEM image), as well as in the degree of graphitization produced (Raman map), which was conducted with higher quality material at the axis of the beam on each line.

Figure 4 shows Raman maps of the LIG materials that are fabricated at four different conditions, specified above each map. The Raman maps were taken in two lines perpendicular to the laser scan direction in order to illustrate the material quality change in regard to the periodicity of the material transformation. The quality of defective graphene can be evaluated using the I_D_/I_G_ and I_2D_/I_G_ ratios, as well as the widths of the peaks [45,46]. Increasing the defect concentration produces an increase in the D peak intensity and of the peak widths, as well as a decrease in the 2D peak intensity; thus, with defects, I_D_/I_G_ increases, while I_2D_/I_G_ decreases. For highly defective samples, the 2D peak was weak and the I_D_/I_G_ reaches values of around 1. For even higher defect concentrations, as in graphene oxide or amorphous carbon, the I_D_/I_G_ begins to decrease due to the reduction in C=C bonds.

In Figure 4a, the I_D_/I_G_ ratio for every point is shown. Darker colors represent more graphitic materials and lighter colors more defective areas. In Figure 4b, the I_2D_/I_G_ ratio is shown. In this case, lighter areas represent higher graphenic quality, with narrow and well-resolved 2D peaks, while darker areas represent lower quality regions. These maps correlate exactly with the periodicity of the laser-induced lines.

In Figure S2 (see Supplementary Materials), typical spectra corresponding to the most defective (a) and less defective (b) points in each sample are shown. The spectra in Figure S2a (i.e., more defective points) are quite similar in all cases, with wide peaks and a very low 2D peak intensity, thus indicating high defect concentrations and high C sp^3^ content. In Figure S2b, the peaks are importantly narrowed and the 2D peak is very well defined—except for sample S-1.8-25, which is obtained with the least IR laser power. Indeed, the I_D_/I_G_ and I_2D_/I_G_ images for this S-1.8-25 sample (Figure 4c) are quite uniform, revealing the homogeneity of the sample transformation but also its poor quality due to the small degree of graphitization. When analyzing I_2D_/I_G_ and I_D_/I_G_ ratios in more detail, especially the peak widths in Figure S2c,d, the best graphene quality corresponds to the S-2.0-45 sample. The D and G peak widths of this sample (around 70 cm^−1^) and an I_2D_/I_G_ ratio close to 0.5 correspond to very high quality multilayer graphene [47,48]. In this situation, the high intensity of the D peak (I_D_/I_G_ around 0.5–0.6) indicates that the defects originated by the edges rather than by the point defects (such as functional groups and C sp^3^) [49]. Therefore, we can describe the sample as being formed by low defective multilayer graphene nanoflakes. Besides the evaluation of the quality of the best and worse points, a decisive aspect is the fraction of high-quality (i.e., low-defective) graphene along the areas that were transformed by the laser. From the images of I_D_/I_G_ (the darker, the better) and I_2D_/I_G_ (the brighter, the better) in Figure 4a,b, it is also clear that the S-2.0-45 sample is the best regarding the fraction of high-quality graphene (Figure 4d). Again, from Figure 4a, in the case of the S-2.2-65 and S-2.4-85 samples, the I_D_/I_G_ ratio is lower than in the case of the S-1.8-25 sample, which is due to the higher level of graphitization for the higher power values. However, their I_D_/I_G_ ratio is higher than the one corresponding to sample S-2.0-45. This is because, despite the higher level of graphitization there is also the excessive removal of material and the collapse of pores that is occurring, as observed before (SEM images, Figure 3) under the two highest power values. The structural degradation of the material translates into the introduction of defects that increase the value of the I_D_/I_G_ ratio.

Microsupercapacitors were fabricated using each pair of the laser conditions, as described in Figure 3 and Figure 4, to investigate the effect of the laser parameters on the electrochemical performance and its correlation with the morphology, which is characterized by the SEM images and the material quality that was assessed by the Raman spectroscopy.

**Figure 5:**
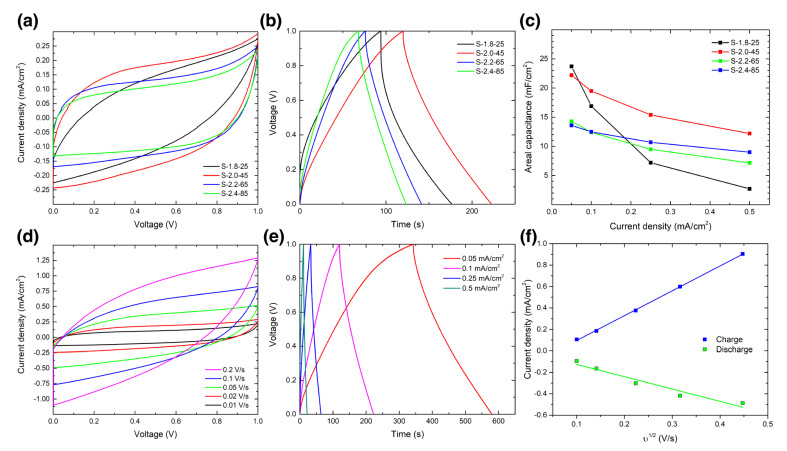
Electrochemical performance of the fabricated MSCs at different laser conditions in the PVA gel electrolyte, 1M H_2_SO_4_. (**a**) Cyclic voltammetry (CV) graphs of the four selected MSCs at a 0.02 V/s scan rate. (**b**) Galvanostatic charge discharge (GCD) graphs for the four selected MSCs at a 0.1 mA/cm^2^ current density. (**c**) Areal capacitance of the four different samples calculated from the GCD as a function of the current density. (**d**–**f**) refer to the sample that displays the best performance: S-2.0-45. (**d**) CV graphs at scan rates from 0.01 to 0.2 V/s. (**e**) GCD curves taken at different current densities from 0.05 to 0.5 mA/cm^2^. (**f**) Capacitive current extracted from the CV curves at 0.5 V, as a function of the square root of the scan rate.

In Figure 5a,b, the CV and GCD graphs were measured under a scan rate of 0.02 V/s and a current density of 0.1 mA/cm^2^, respectively, for each sample and are represented together under the same scale to enable the comparison. In addition, the electrochemical impedance spectroscopy (EIS) measurements are provided in Figure S3 (Supplementary Materials). Figure 5c summarizes the calculated areal capacitance values at every current density, as extracted from the charge/discharge measurements. The best performing sample overall was S-2.0-45, as plotted in red in Figure 5a–c. Only at the very low current densities of 0.05 mA/cm^2^ does S-1.8-25 outperform it in terms of areal capacitance, but this metric quickly drops for larger currents in this sample (Figure 5c). Figure 5d–f shows the CV, GCD, and capacitive current of sample S-2.0-45 under all the measured conditions, respectively. The complete graphs of the other samples shown in Figure 5a–c (namely, S-1.8-25, S-2.2-65 and S-2.4-85), plus the remaining fabricated samples (S-1.8-40, S-2.0-60, S-2.2-80 and S-2.4-70) can be found in Figures S4 and S5 (Supplementary Materials). Additionally, in Figure S6, the calculation of the non-diffusive contribution to the capacitance is plotted following Dunn’s method [50]. The current densities obtained at increasing CV scan rates follow an exponential decrease (Figure 5f), thus making clear the power limitation present in these LIG MSC devices [10]. This reduction in the current can be also explained by means of the ion transport in the electrodes, as at lower scan speeds the ions have more time to diffuse through the electrolyte, thus entering into the deeper pores of the electrode. Meanwhile, at faster scan rates, only the more superficial pores are reached, and the effective surface area of the supercapacitor is reduced [51].

## 4. Discussion

**Figure 6:**
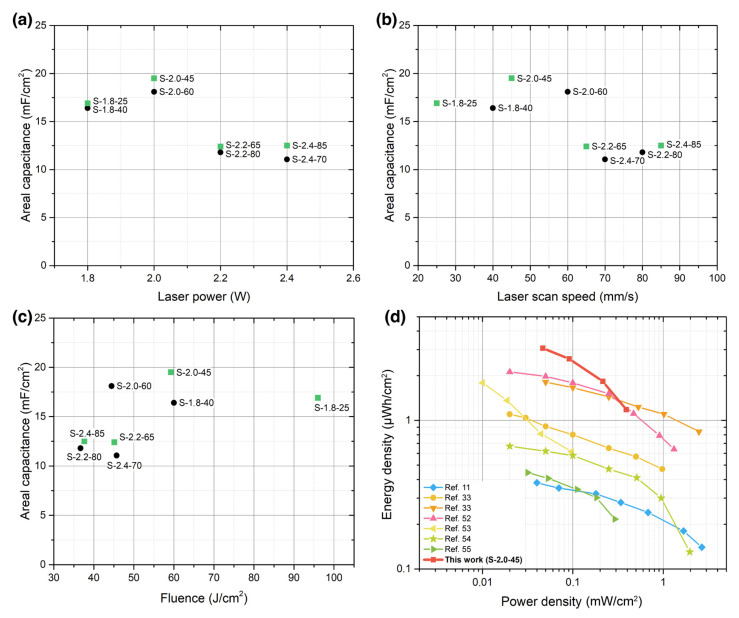
Areal capacitance of the measured microsupercapacitors in the 1M H_2_SO_4_ PVA gel electrolyte at 0.1 mA/cm^2^, as a function of the main laser parameters: (**a**) laser power, (**b**) laser scan speed, and (**c**) laser fluence. The four samples of interest mentioned in Figure 3, Figure 4 and Figure 5 are marked as green squares, while the other four complete the samples mentioned in Figure 2. (**d**) Area-specific Ragone plot, including the device S-2.0-45 and similar devices from the literature.

In order to compare all eight laser conditions, as introduced in Figure 2, and their viability to fabricate the best performing LIG microsupercapacitors, the calculated areal capacitances of each condition are plotted in Figure 6a–c, as well as averaged between the multiple samples fabricated at each condition.

In Figure 6a, the areal capacitance (at 0.1 mA/cm^2^) versus the laser power in watts is shown. The pairs of microsupercapacitors manufactured with the same laser power render very similar capacitance results, regardless of its laser scan speed. This proves that the laser power is the most relevant parameter governing the transformation of the material in regard to achieving the largest capacitance. Plotting the same capacitance data against the laser scan speed (Figure 6b) shows a similar behavior, which is clearly influenced by the laser power.

Regardless of the laser fluence being one of the most used parameters in these pyrolysis methods, we have not clearly observed its influence when plotting the areal capacitance of the fabricated interdigitated microsupercapacitors (see Figure 6c). The conditions with the lowest fluence, S-2.2-80 and S-2.4-85, are in the lowest capacitance range, as are S-2.2-65 and S-2.4-70, which are also with a fluence lower than 50 J/cm^2^. However, S-2.0-60, which has an even lower fluence than those mentioned prior, showed a 40% higher capacitance. The highest capacitances were found in the lowest power samples, i.e., those manufactured at 1.8 and 2 watts.

These results are in agreement with the microstructural SEM images of each LIG condition. As stated before, sample S-2.0-45 was the most homogeneous and with the best porosity distribution, whereby it showed more and had smaller pores of around 2.5 µm, which was in contrast with the bigger pores of the higher power samples. S-2.0-45 was also the one with the highest graphenic quality at the center of each raster line, according to the Raman spectroscopy mapping. It also showed the lowest I_D_/I_G_ ratio and the highest I_2D_/I_G_ ratio of all four samples. Moreover, the material of S-2.0-45 is composed of low defective multilayer graphene nanoflakes, conforming to a continuous porous network. This sample reached a maximum capacitance of 22.2 mF/cm^2^ at 0.05 mA/cm^2^, which is one of the highest values reported in the literature for an all-carbon LIG MSC.

The Ragone plot in Figure 6d situates these results within the comparable literature [11,33,52,53,54,55] in terms of areal energy density and areal power density, which are common metrics to benchmark and compare with respect to other energy storage devices. In order to establish a fair comparison, Figure 6d only includes bare LIG devices, which were fabricated on polyimide substrates and without any addition of heteroatoms or pseudocapacitive nanoparticles. The best device obtained in this work reaches 3.07 µWh/cm^2^ and 0.0462 mW/cm^2^ at 0.05 mA/cm^2^, which is higher than similar devices in the literature—even those doped with N or B atoms [33,52]. The energy density decreased down to 1.18 µWh/cm^2^ at 0.5 mA/cm^2^, which is 40% less than the measurements found at the lowest current density. Further, it is a decrease that, while being steeper than those found in other cited devices, still keeps the device as one of the best. In addition, these results are even better if it is taken into account that this LIG MSC was undoped from any pseudocapacitive element.

A parameter that has a great impact on the capacitance of these electrodes and devices is the thickness of the LIG layers, as well as its relation to the thickness of the original substrate and precursor. Most studies use a 125–127 µm thick polyimide tape, as this is how it is manufactured conventionally, but only transform around 50 µm into a usable LIG material [11,19,22,52,54,55,56,57,58,59,60]. By using 60 µm polyimide tape, we are able to reduce the total volume of the used material, reaching much a higher active material to total volume ratio, while maintaining the same LIG thickness and, therefore, the same electrochemical performance. This has implications in regard to sustainability as less waste is produced, but is also relevant in terms of its integration. Apart from the lateral miniaturization of the devices, the shrinking of the thickness will maximize its ability to better adapt to the mechanical loads and to be more conformal to the required bending conditions. On top of that, thin devices also allow one to stack them into higher voltage or higher current modules in thin and small packages, which can be implemented on the skin or clothing of the users in a non-intrusive and comfortable way [61,62,63].

To summarize, the fabrication of thin LIG microsupercapacitors will further boost their use in flexible and wearable electronics [64,65], such as in the healthcare industry regarding power sensors [53,58] or in other medical devices. They could even be used in single-use, disposable devices [57,66]. Furthermore, once the fabrication of thin bare LIG MSCs with optimal properties is well developed and understood, the combination of LIG substrates with other pseudocapacitive elements will constitute the next step in greatly increasing the capacitance of the devices—as has been demonstrated in recent years, whereby elements such as MoS_2_ [67,68], ZnP [69] or Co_3_O_4_ nanoparticles have been added [70].

## 5. Conclusions

In conclusion, we have investigated the relation between the LIG structural morphology, graphenic quality, and electrochemical performance on a set of laser-induced graphene microsupercapacitors that were fabricated on 60 µm thick Kapton film. The selected laser conditions cover all the possible range of power and scan speed that transformed the material, while keeping its structural continuity for the used Kapton. For their application as supercapacitor electrodes, we have found that the most relevant parameter in LIG formation on thin layers is the laser power, as this parameter dominated in impact over laser fluence or scan speed. The devices fabricated at a 2 W laser power (S-2.0-45) showed more homogeneous and smaller pores, of around 2.5 µm, as well as the highest material quality, as inspected through Raman spectroscopy mapping with an I_2D_/I_G_ ratio close to 0.5 and a similar I_D_/I_G_ ratio. Both metrics indicated that the LIG material that was transformed at 2 W was composed up of high-quality, low-defective, multilayer graphene nanoflakes in comparison with the other laser conditions, which produce lower quality materials. This explains the high electrochemical performance of sample S-2.0-45, with an average areal capacitance of 22.2 mF/cm^2^ at 0.05 mA/cm^2^—which is among the highest reported in the literature for a LIG MSC without any pseudocapacitive material added. This device also reaches an energy density of 3.07 µWh/cm^2^ and a power density of 0.0462 mW/cm^2^ at 0.05 mA/cm^2^, which is comparable to other LIG MSCs, even those doped with heteroatoms. In all, this work has demonstrated that it is possible to obtain high-quality and high-performing laser-induced graphene on thin layers of polyimide in a single laser pass, which is suitable for flexible applications.

## Figures and Tables

**Table 1:** Summary of the laser conditions of the eight fabricated samples, thereby showing its name, power, scan speed, and calculated laser fluence value. Samples marked with (*) provide an equally spaced sequence of increasing values of power and speed.

Sample Name	Power (W)	Scan Speed (mm/s)	Fluence (J/cm^2^)
S-1.8-25 (*)	1.8	25	96
S-1.8-40	1.8	40	60
S-2.0-45 (*)	2.0	45	59.3
S-2.0-60	2.0	60	44.4
S-2.2-65 (*)	2.2	65	45.1
S-2.2-80	2.2	80	36.7
S-2.4-70	2.4	70	45.7
S-2.4-85 (*)	2.4	85	37.6

## Data Availability

Data is available upon request.

## Supplementary Materials

The following supporting information can be downloaded at: https://www.mdpi.com/article/10.3390/nano13050788/s1, Table S1—Summary of the common and specific traits, advantages, and disadvantages of the available laser methods to fabricate LIG electrodes; Figure S1—Electrical resistance of the laser induced graphene samples studied in this work. Each point represents the average value of three samples fabricated under the same conditions including the calculated standard deviation (y-axis error bars). The samples represented by the green circles are the four ones fabricated under the conditions that correspond to equally spaced power and scan speed (0.2 W and 20 mm/s between samples), focus on this work (see Figure 2 and Table 1 in the main text); Figure S2—(a,b) Stacked Raman spectra of the four selected samples, at the worst graphenic quality point (a) and the best graphenic quality point (b) for each sample. (c) Raman peak intensity ratios for each sample, I_D_/I_G_ and I_2D_/I_G_. (d) FWHM of the D and G peaks for every sample; Figure S3—Electrochemical Impedance Spectroscopy plot of the four main samples. The inset shows the EIS data at high frequencies; Figure S4—Cyclic voltammetry graphs of all samples at different scan speeds, from 0.01 V/s to 0.2 V/s: (a) S-1.8-25, (b) S-2.0-45, (c) S-2.2-65, (d) S-2.4-85, (e) S-1.8-40, (f) S-2.0-60, (g) S-2.2-80, (h) S-2.4-70; Figure S5—Galvanostatic charge discharge graphs of all samples at different current densities, from 0.05 mA/cm^2^ to 0.5 mA/cm^2^: (a) S-1.8-25, (b) S-2.0-45, (c) S-2.2-65, (d) S-2.4-85, (e) S-1.8-40, (f) S-2.0-60, (g) S-2.2-80, (h) S-2.4-70; Figure S6—Cyclic Voltammetry curves of sample S-2.0-45 at 0.02 V/s (a), and 0.1 V/s (b). Non-diffusive contribution to the capacitance calculated from CVs (0.01 to 0.1 V/s) following Dunn’s method.
