# Supplementary material for: Laser-Induced Graphene Microsupercapacitors: Structure, Quality, and Performance
Source: Nanomaterials (Basel). 2023 Feb 21;13(5):788. doi: 10.3390/nano13050788 (PMC10005378; doi:10.3390/nano13050788)
Supplement: Supplementary file 1 [file nanomaterials-13-00788-s001.zip › nanomaterials-2202922-supplementary.pdf]

Supplementary material

Laser-induced Graphene Microsupercapacitors: Structure, Quality, and Performance

Andres Velasco [1,2], Yu Kyoung Ryu [1,*], Assia Hamada [1], Alicia de Andrés [3], Fernando Calle [1,2] and Javier Martinez [1,4,*]

[1] Instituto de Sistemas Optoelectrónicos y Microtecnología, Universidad Politécnica de Madrid,
Av. Complutense 30, 28040 Madrid, Spain
[2] Departamento de Ingeniería Electrónica, Escuela Técnica Superior de Ingenieros de Telecomunicación,
Universidad Politécnica de Madrid, Av. Complutense 30, 28040 Madrid, Spain
[3] Instituto de Ciencia de Materiales de Madrid, Consejo Superior de Investigaciones Científicas,
C/Sor Juana Inés de la Cruz 3, Cantoblanco, 28049 Madrid, Spain
[4] Departamento de Ciencia de Materiales, Escuela Técnica Superior de Ingenieros de Caminos, Canales y Puertos, Universidad Politécnica de Madrid, C/Profesor Aranguren s/n, 28040 Madrid, Spain
* Correspondence: y.ryu@upm.es (Y.K.R.); javier.martinez@upm.es (J.M.)

Laser method	Advantages	Disadvantages	Specific features	Common features
CO_2 laser	Low cost<br>Commercial<br>Simple setup<br>High power	Lower resolution	Continuous wave without addition of optical choppers<br>Photothermal mechanism	Large variety of transformable precursors, including bio-based/biodegradable<br>Simultaneous material transformation and device patterning in a single step<br>Fast processing<br>High tunability through the modulable parameters and available precursors enable graphene with a wide range of structures and applications
UV/visible laser	More dense structures achievable<br>Access to the pulse duration and repetition parameters	Higher resolution	Continuous and pulsed mode<br>Photothermal and photochemical mechanisms	

Table S1 – Summary of the common and specific traits, advantages, and disadvantages of the available laser methods to fabricate LIG electrodes.

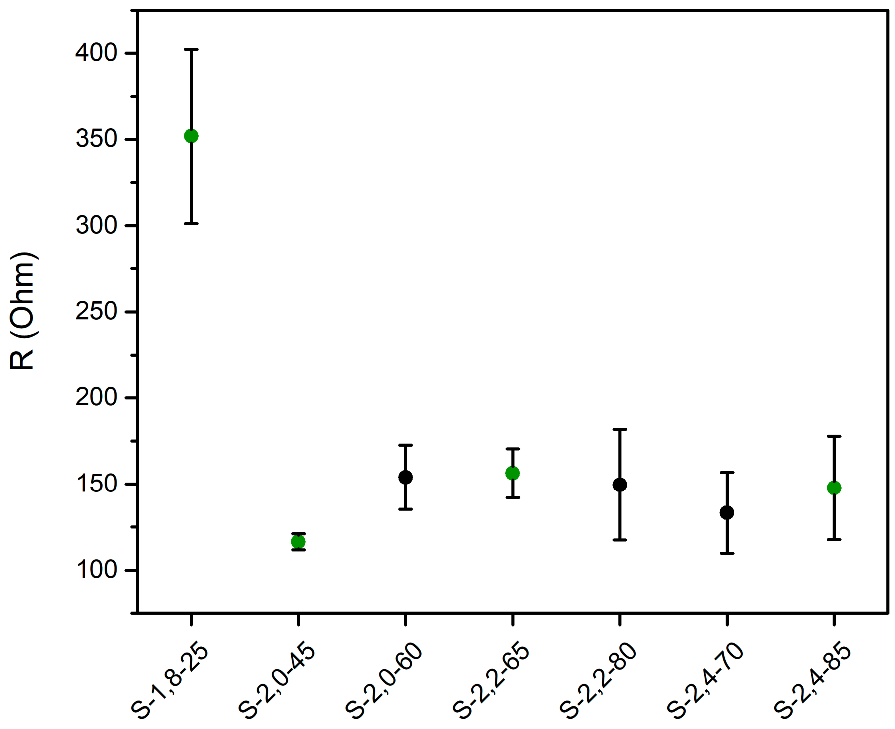

Figure S1 – Electrical resistance of the laser induced graphene samples studied in this work. Each point represents the average value of three samples fabricated under the same conditions including the calculated standard deviation (y-axis error bars). The samples represented by the green circles are the four ones fabricated under the conditions that correspond to equally spaced power and scan speed (0,2 W and 20 mm/s between samples), focus on this work (see figure 2 and table 1 in the main text).

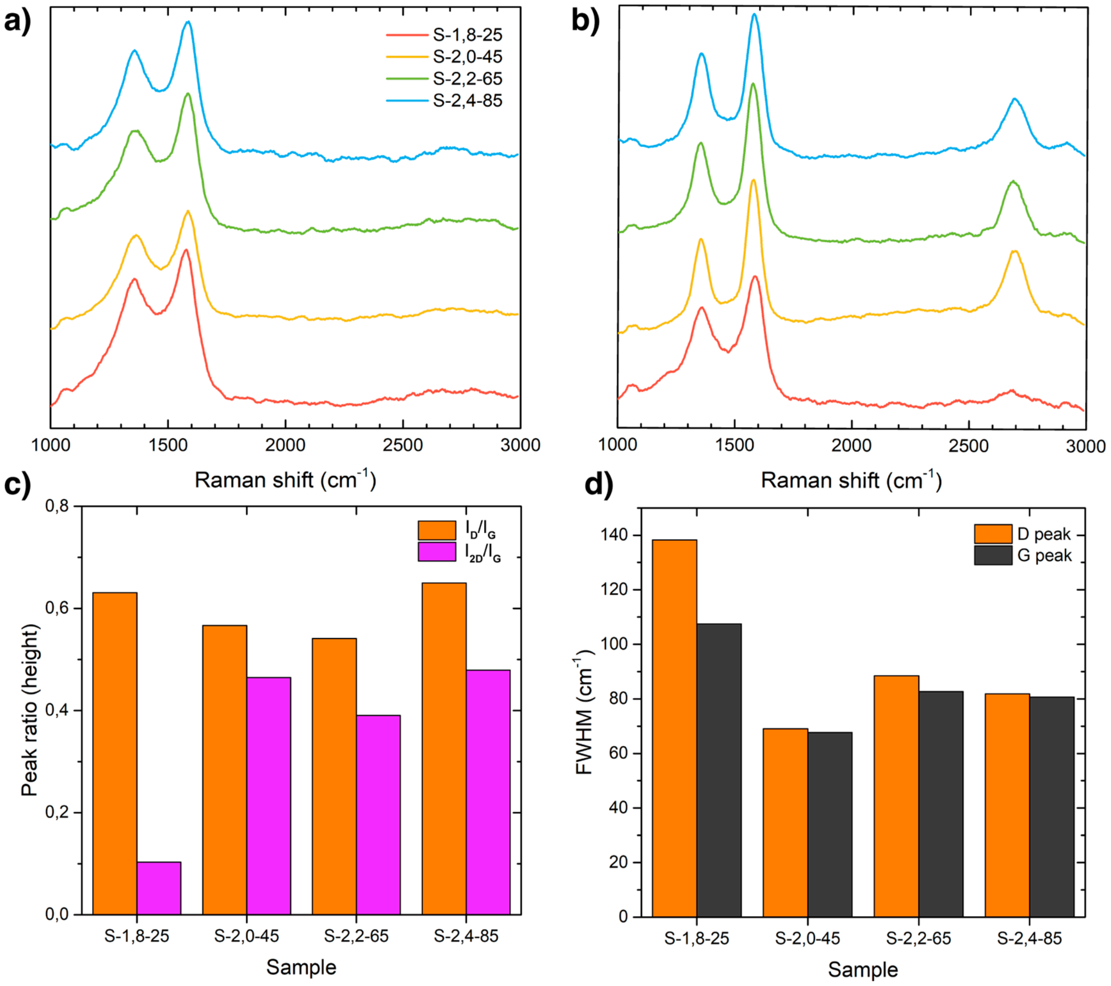

Figure S2 – (a, b) Stacked Raman spectra of the four selected samples, at the worst graphenic quality point (a) and the best graphenic quality point (b) for each sample. (c) Raman peak intensity ratios for each sample, I_D/I_G and I_{2D}/I_G. (d) FWHM of the D and G peaks for every sample.

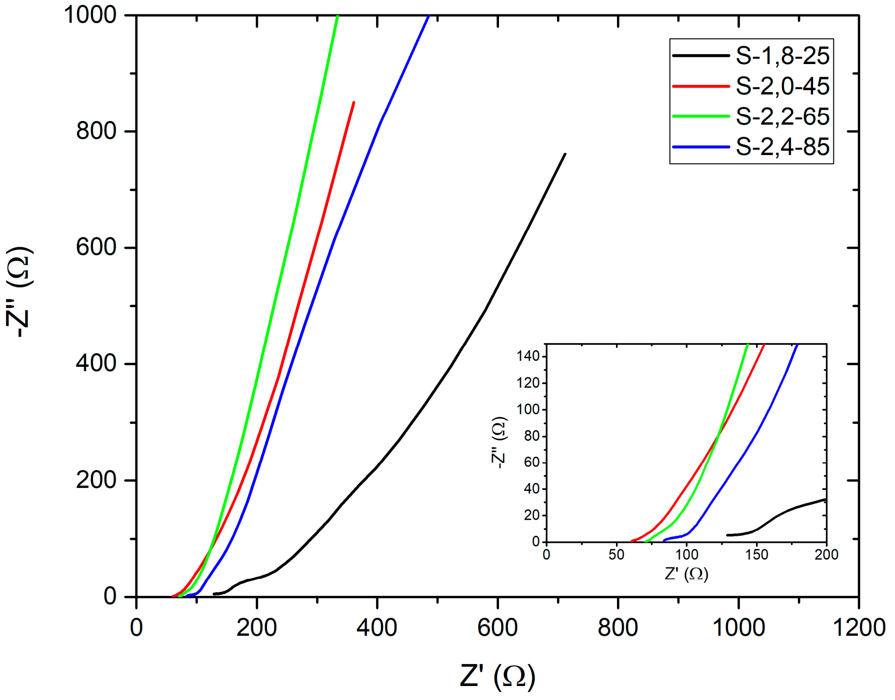

Figure S3 – Electrochemical Impedance Spectroscopy plot of the four main samples. The inset shows the EIS data at high frequencies.

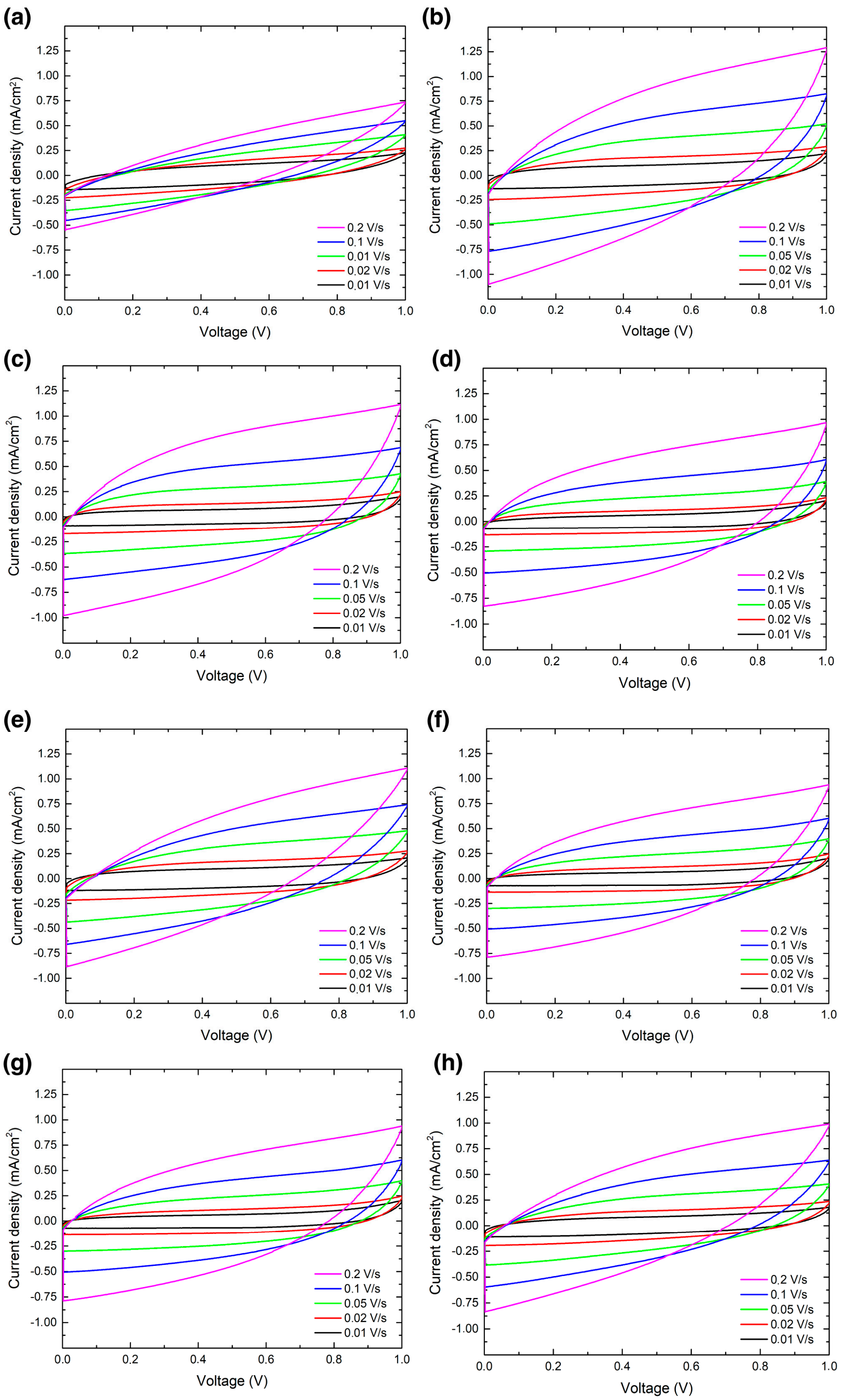

Figure S4 - Cyclic voltammetry graphs of all samples at different scan speeds, from 0,01 V/s to 0,2 V/s: (a) S-1,8-25, (b) S-2,0-45, (c) S-2,2-65, (d) S-2,4-85, (e) S-1,8-40, (f) S-2,0-60, (g) S-2,2-80, (h) S-2,4-70

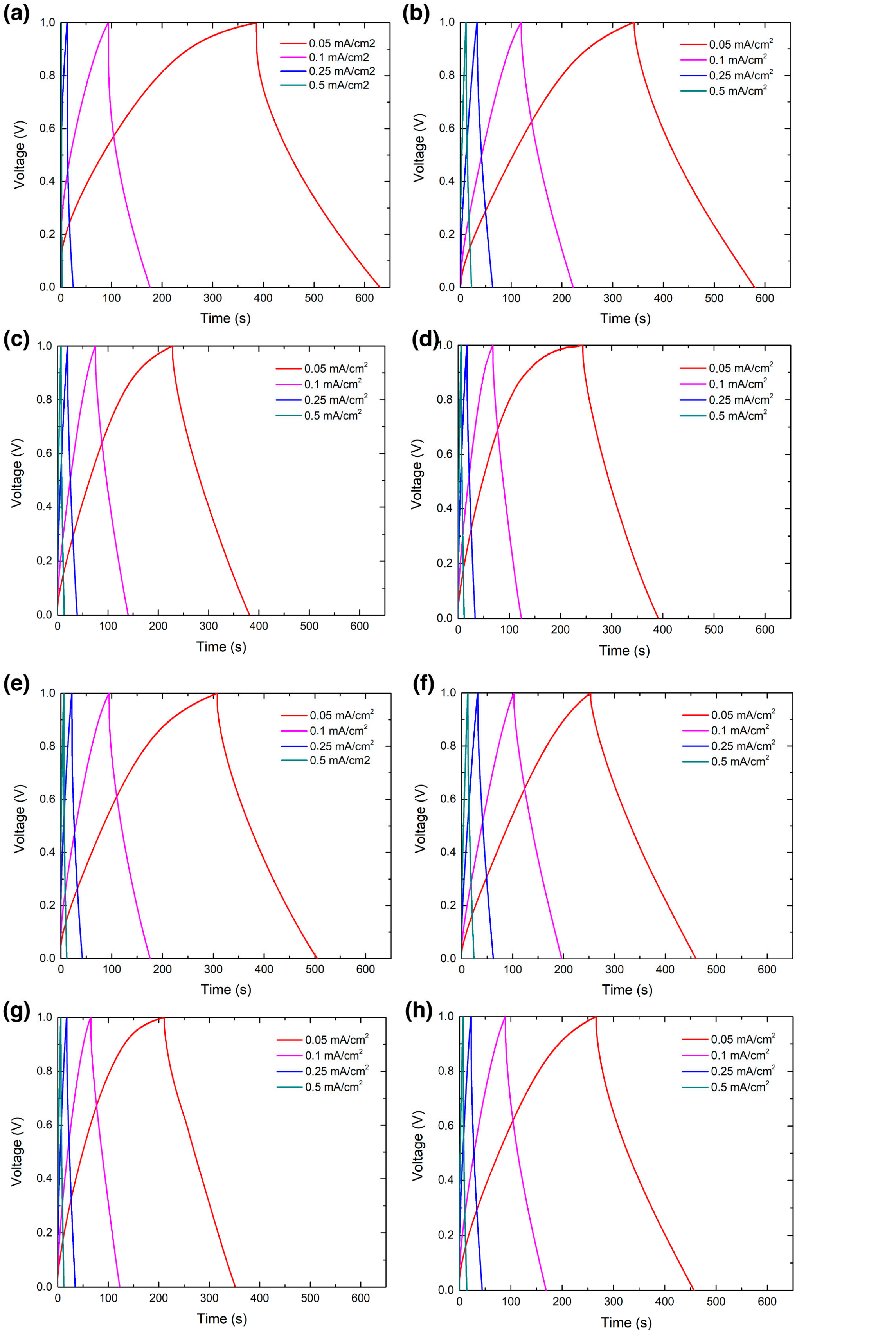

Figure S5 – Galvanostatic charge discharge graphs of all samples at different current densities, from 0,05 mA/cm^2 to 0,5 mA/cm^2: (a) S-1,8-25, (b) S-2,0-45, (c) S-2,2-65, (d) S-2,4-85, (e) S-1,8-40, (f) S-2,0-60, (g) S-2,2-80, (h) S-2,4-70.

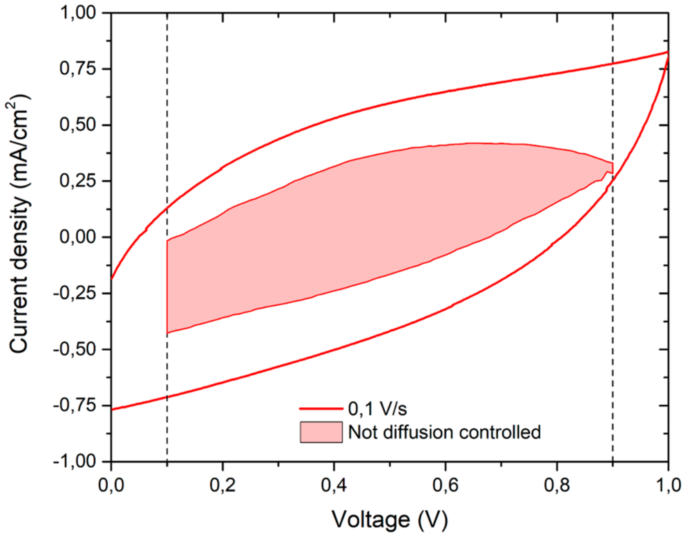

Figure S6 – Cyclic Voltammetry curves of sample S-2,0-45 at 0,02 V/s (a), and 0,1 V/s (b). Non-diffusive contribution to the capacitance calculated from CVs (0,01 to 0,1 V/s) following Dunn's method.
